# Sonographic prediction of fetal main pulmonary artery (MPA) Doppler indices of lung maturity and neonatal respiratory distress syndrome (RDS) development

**DOI:** 10.1007/s40477-023-00780-w

**Published:** 2023-04-20

**Authors:** Hend Galal Eldeen Mohamed Ali Hassan, Mona Ali Mohamed Ali Nagi, Asmaa Magdy Mohamed Salama, Mohamed Omar Abd El Aal Dawoud, Ghalia Galal Elgendy, Ahmed S. Abdelrahman

**Affiliations:** 1 Diagnostic and Interventional Radiology Department, Faculty of Medicine, Ain Shams University, 38 Ramsis Street, Abassia, Nasr City, Cairo, 11765 Egypt; 2 Pediatric Department, Faculty of Medicine, Ain Shams University, Cairo, Egypt; 3 Obstetric and Gynecology Department, El Sheikh Zayed Al Nahyan Hospital, Cairo, Egypt; 4 Technology of Radiology and Medical imaging program, Faculty of Applied Health Sciences Technology, Galala University, Suez, Egypt

**Keywords:** Main pulmonary artery Doppler, Lung maturity, Respiratory distress syndrome

## Abstract

**Purpose:**

The aim of this study is to highlight the predictive role of perinatal fetal main pulmonary artery (MPA) Doppler measurements in neonatal respiratory distress syndrome development. Respiratory distress syndrome (RDS) is one of the lead causes of neonatal respiratory distress as well as neonatal death. Thus, it seems logic to evaluate fetal lung maturity before labour.

**Methods:**

The study is a prospective cohort study performed in tertiary hospital over a period of one-year duration. 70 pregnant ladies between 34 and 38 weeks of gestation were referred for fetal echo, when pregnancy was considered a high risk. A trained radiologist using dedicated ultrasound machine with updated obstetric and fetal echo software performed the fetal echo. Doppler mode and curvilinear probe of 5.7 MHz transducer. Pediatric neonatologist observed the neonatal outcome post-natally.

**Results:**

A total of 70 pregnant patients with risk factors underwent fetal echo, 26/70 (37.1%) were diagnosed with RDS conforming to the neonatal criteria. The mean acceleration time/ejection time ratio (At/Et ratio) of the fetal pulmonary artery was significantly reduced in fetuses that subsequently developed RDS than those without RDS. Contrarily, the mean pulsatility index (PI), resistance index (RI), and peak systolic velocity (PSV) of the fetal pulmonary artery were significantly high in fetuses who later developed RDS than in those who did not.

**Conclusion:**

Fetal MPA Doppler measurements have a major role in anticipating the development of neonatal RDS in preterm and early term neonates.

## Introduction

Neonatal respiratory distress syndrome (RDS) implies a respiratory deterioration that presents at or shortly after birth due to a deficiency of natural phospholipid namely pulmonary surfactant, which is essential in decreasing the surface tension inside the alveoli thus, preventing alveolar collapse. RDS is one of the main causes of neonatal morbidity and mortality [1].

The respiratory system is one of the delayed fetal systems to functionally mature for extra- uterine life accommodation. Therefore, RDS is mainly a prematurity disease, its incidence and severity have a direct relationship with the gestational age [2].

As RDS is one of the leading causes of neonatal morbidity and mortality, multiple tests have been used to predict the risk of RDS and thus aiding the obstetricians care in deciding delivery timing and modality. However, most of these tests require amniocentesis which is although a minimally invasive procedure still has multiple pregnancy risks including preterm premature rupture of membranes, preterm labor, placental abruption, feto-maternal haemorrhage, fetal injury, and (rarely) fetal or even maternal death. So, searching for non-invasive methods using ultrasound to estimate fetal lung maturity have been long tried (such as measurements of gestation age, placental grading, and estimated fetal weight) but haven’t been of evident value in clinical practice [3].

As the lungs develop throughout gestation, so does the pulmonary vasculature, where both sum of proximal pulmonary arteries diameter increases and maturation of vascular smooth muscle progresses, and the pulmonary arterial vascular resistance decreases slightly, resulting in progressive increase in pulmonary blood flow [4].

The hypothesize of this study is that non-invasive interrogation of the pulmonary vasculature provides significantly valuable information about functional pulmonary maturity.

Doppler velocimetry provides a simple and non-invasive method to assess the fetal pulmonary circulation. Several investigators have used Doppler velocimetry to measure fetal pulmonary blood flow in pulmonary artery and peripheral branches, but the rate of accurate Doppler recordings is moderate (77–84%), and the results are widely disparate [5], because as gestation progresses, the Doppler velocity waveform in the fetal MPA change. Many trials have studied the relation between fetal pulmonary artery Doppler waveforms and fetal pulmonary hypoplasia [6]. Fetal pulmonary artery acceleration time/ejection time ratio has a strong correlation with the increasing fetal gestational age [7].

In our study, we used the measurement of fetal MPA Doppler indices and correlate the results with the consequent neonatal RDS development.

The aim of our study is to measure the fetal MPA Doppler measurements and assess its role in anticipating neonatal RDS development in preterm and early term neonates and assess the outcome of prenatally detected MPA abnormalities suggestive of RDS and their effect in the neonatal survival.

## Materials and methods

The study is a prospective study that was conducted in tertiary level hospitals (Ain Shams university and Alsheikh Zayed Alnahian), Cairo, Egypt from April 2020 to April 2021.

This study was approved by the local institutional review board (IRB) which is the Research Ethics Committee of the Faculty of Medicine at Ain shams University in Egypt (FWA 000017585) on March 2020; Reference Number of approval: R 14/2020. An informed written consent was obtained from all participants in this study.

Ladies admitted to the delivery unit between 34 and 38 + 6 gestational weeks with an accurate GA (defined as dating by a certain last menstrual period or by first-trimester ultrasound), either in active labor or scheduled for selective CS, were consecutively enrolled in the study, only fetuses delivered within 24–48 h of admission were included.

Pregnant ladies were referred consecutively, where pregnancy was considered as high risk for developing neonatal RDS. The standard risk factors were either due to gestational, familial or fetal causes e.g., gestational diabetes mellitus (GDM), premature rupture of membrane, placenta previa or abruption, oligo or polyhydramnios were included in our study.

In contrary, pregnant ladies with twin or more gestation, known fetal chromosomal or major structural abnormality, Prior antenatal corticosteroid administration were excluded. Fetuses with structural anomalies after delivery were also not enrolled in our study.

After acquiring an informed consent, maternal age as well as parity were documented then an ultrasound was performed. Doppler study of the fetal pulmonary artery and fetal cardiac scanning was done by a single radiologist with more than 5 years’ experience of fetal imaging speciality using a suitable updated ultrasound equipped machine with convex transducer having wide frequency range (ACUSON Juniper Siemens USA). To improve the chance of finding subtle Doppler changes, the highest frequency (5.7 MHz), a single acoustic focal zone, and a narrow image field were used.

After obtaining the fetal biometry, estimated fetal weight and amniotic fluid index, the fetal echo software switched on to examine the fetal heart in a systematic manner (the four-chamber view, the outflow tracts and the three-vessel view). At the axial view of the thorax, with the fetus stable without fetal breathing movements, the ultrasound specialist traced the MPA until the midpoint between the pulmonary valve and the bifurcation of the right and left branches. The pulsed Doppler sample gate was adjusted to 3 mm and the angle of insonation was kept at or around 15°.

**Fig. 1:**
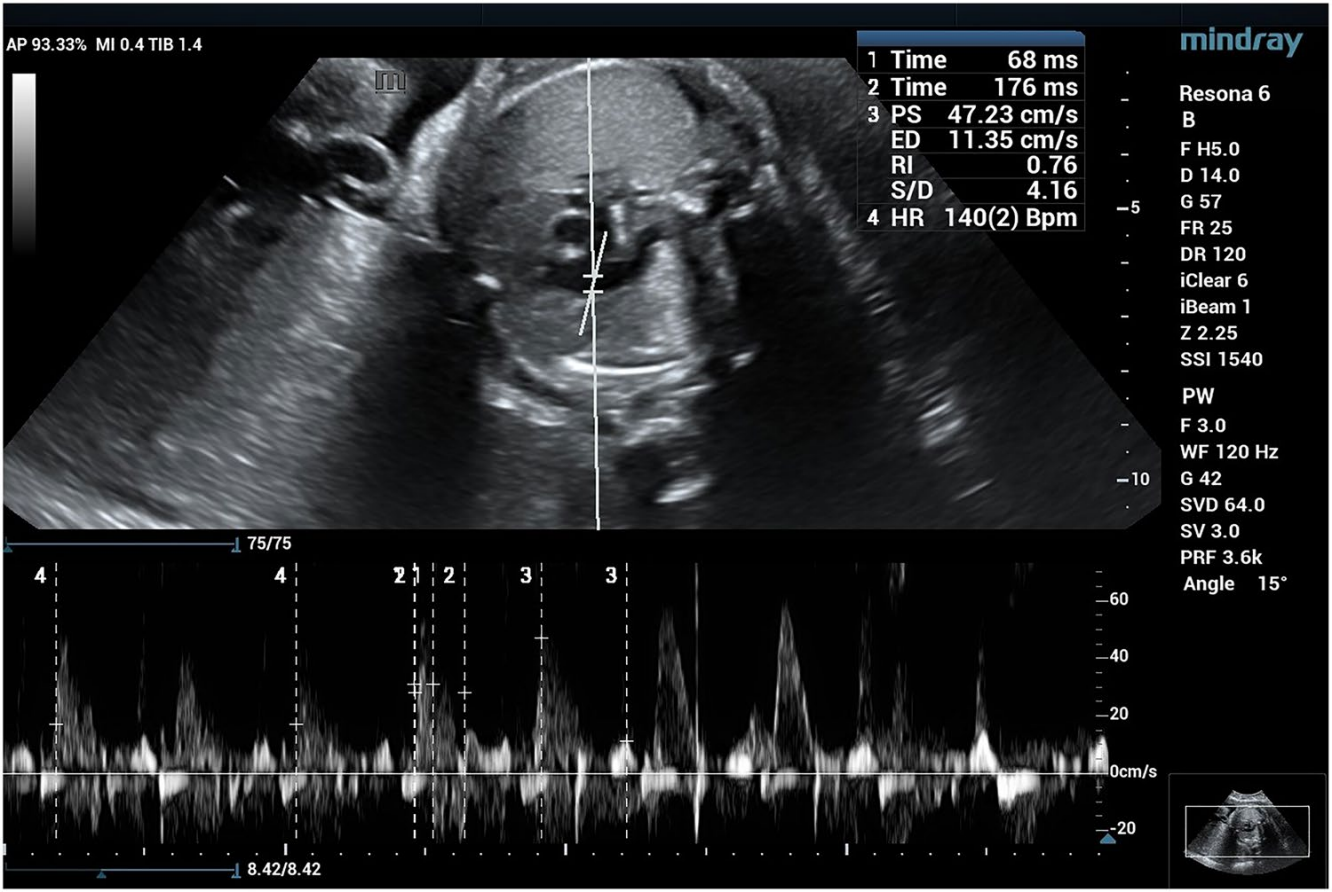
The optimal fetal MPA waveform shape and parameter with Doppler velocity measurements were manually tracked including systolic/diastolic (S/D) ratio, resistance index (RI), PSV and acceleration T, ejection T, AT/ET ratio is manually calculated

Doppler gain and scale were tailored for optimizing the velocity waveform display clearly showing the peak systolic velocity (PSV) and early diastolic notch. The MPA Doppler waveform showed with its distinguished shape (sharp systolic peak blood flow with a needle-like appearance, commonly referred to as a 'spike and dome' pattern). A small notch of reversed flow is also seen at the end of the systole [8]. The characteristic shape of MPA waveform is important to differentiate it from the wave of the ductus arteriosus, which is rounded, fuller and triangular in shape with greater diastolic flow [9]. After the optimum fetal MPA waveform was obtained, relevant Doppler velocity variables were manually traced three times and the average was taken. The variables included the systolic/diastolic (S/D) ratio, pulsatility index (PI), resistance index (RI), PSV and the At/Et ratio. To obtain the At/Et ratio, the time interval from the beginning of the ventricular systole to the achievement of peak velocity (At) was divided by the time interval from the start to the end of ventricular systole (Et) (Fig. 1).

Preterm birth is defined as any birth before 37 weeks of gestation. Extremely preterm is delivery less than 28 weeks, whereas very preterm is 28 to < 32 weeks. Moderate or late preterm is 32 to < 37 completed weeks of gestation [10].

### Diagnosis of post-natal

Immediately after delivery, the type of delivery was documented as well as the sex of the neonate by the obstetrician from the research team. A single paediatric neonatologist, from the research team, to whom the fetal MPA Doppler measurements were obscured, handled the neonate. Neonatal birth weight (NBW) and Apgar score (at 1 and 5 min) were recorded. The RDS diagnosis was established upon clinical signs of respiratory difficulty (tachypnea, retractions and/or nasal flaring), supplemental oxygen requirement of 0.4 or greater for at least 24 h and typical chest X-ray findings with reticulogranular patterns, air bronchograms and ground glass appearance (Model: XS-1A, Chongqing Hualun Medical Instrument, China). The duration of neonatal intensive care admission was also recorded.

Statistical analysis Data was described as Statistical Package for Social Science (IBM Corp. Released 2017. IBM SPSS Statistics for Windows, Version 25.0. Armonk, NY: IBM Corp.) was used for data analysis. Parametric continuous data were reported as mean ± standard deviation (SD). Median and range were used to express the non-parametric data. Qualitative data were described as frequency and percentage. Independent same *t* test was conducted to compare continuous variable. Chi-square test was performed to compare categorical data. Mann–Whitney *U* test was used to compare ordinal data. Receiver operator characteristic (ROC) curve analysis was performed to set the optimal cut off value. The sensitivity, specificity, PPV and NPV were then calculated. A Multinomial logistic regression model was performed to set the predictor of RDS after adjustment of gestational age. *p* value < 0.05 was considered statistically significant.

## Result

Our study included 70 fetuses (70 mothers); the mean maternal age was 31.56 ± 6.31 years (range 20–43 years), 30/70 (42.9%) were nullipara and 40 (57.1%) were multipara. The maternal risk factors were diabetes mellitus (11), hypertension and preeclampsia (12), premature rupture of membrane (13), placenta previa (11), diabetes mellitus (DM) with premature rupture of membrane (PROM) (14) and hypertension (HTN)with PROM (9). The mean gestational age at delivery was 35.41 ± 0.97 weeks (range 34–38). 30 (42.9%) neonates were early preterm (34 to 35 weeks), 25 (35.7%) were late preterm (35 + 1 to 36 weeks) and 15 (21.4%) were early term (36 + 1 to 38 weeks). 26/70 (37.1%) were diagnosed with RDS according to the neonatal criteria (Table 1).

**Table 1:** Maternal and neonatal clinical feature

Mean maternal age (range)	31.56 ± 6.31 (20–43)
Parity (no. (%))	
Nulli	30 (42.9%)
Multi	40 (57.1%)
Risk factor (no. (%))	
DM	11 (15.7%)
HTN and preeclampsia	13 (18.6%)
PROM	12 (17.1%)
Placenta previa	11 (15.7%)
DM + PROM	14 (20%)
HTN + PROM	9 (12.9%)
Delivery (no. (%))	
Vaginal	29 (41.4)
CS	41 (58.6%)
Mean gestational age in weeks (range)	35.41 ± 0.97 (34–38)
Age by category (no. (%))	
Early preterm	30 (42.9%)
Late preterm	25 (35.7%)
Early term	15 (21.4%)
Gender age by category (no. (%))	
Male	36 (51.4%)
Female	34 (48.6%)
Neonatal RDS (no. (%))	26 (37.1%)

The maternal, neonatal data in addition to the fetal ultrasound findings in neonate diagnosed with RDS and those without RDS are presented in (Table 2). Neonate that developed RDS had a significantly lower GA at delivery (*p* = 0.026), lower estimated fetal weight on ultrasound (*p* < 0.001), lower birth weight (*p* < 0.001), and lower amniotic fluid index (*p* = 0.013). Neonate with RDS had also significantly lower 1- and 5-min Apgar scores (*p* < 0.001), a higher number of 5-min Apgar score less than 7 (*p* < 0.001) and were more commonly admitted to neonatal intensive care unit (*p* < 0.001). No statistically significant difference between both groups regarding maternal age, parity, risk factors, mode of delivery and neonatal gender.

**Table 2:** Comparison between fetuses with and without neonatal RDS

	Fetuses with RDS (*n* = 26)	Fetuses without RDS (*n* = 44)	*p* value
Maternal data			
Maternal age (mean)	31.54 ± 6.95	31.57 ± 5.99	0.985^a^
Nulli (no. (%))	10 (38.5%)	20 (45.5%)	0.568^b^
Risk factor (no. (%))	5 (19.2%)	6 (13.6%)	0.702^b^
DM	3 (11.5%)	10 (22.7%)	
HTN and preeclampsia	6 (23.1%)	6 (13.6%)	
PROM	3 (11.5%)	8 (18.2%)	
Placenta previa	6 (23.1%)	8 (18.2%)	
DM + PROM	3 (11.5%)	6 (13.6%)	
HTN + PROM			
Mode of delivery (no. (%))			0.164^b^
VD	8 (30.8%)	21 (47.7%)	
CS	18 (69.2%)	23 (52.3%)	
Mean GA at delivery (weeks)	34.90 ± 0.67	35.71 ± 1.00	< 0.001*^a^
Age by category (no. (%))			0.026*^b^
Early preterm	16 (61.5%)	14 (31.8%)	
Late preterm	8 (30.8%)	17 (38.6%)	
Early term	2 (7.7%)	13 (29.5%)	
US measurements			
EFW mean (kg)	2.122 ± 0.301	2.808 ± 0.452	< 0.001*^a^
Mean AFI (cm)	12.31 ± 5.59	15.59 ± 4.99	0.013*^a^
AF category (no. (%))			0.041*^b^
Oligohydramnios	8 (30.8%)	4 (9.1%)	
Average	16 (61.5%)	31 (70.5%)	
Polyhydramnios	2 (7.7%)	9 (20.5%)	
Neonatal data			
Male (no. (%))	15 (57.7%)	21 (47.7%)	0.420^b^
NBW mean (kg)	2.165 ± 0.305	2.850 ± 0.452	< 0.001*^a^
Apgar 1 min (median (range))	6 (4–7)	7 (6–10)	< 0.001*^c^
Apgar 5 min (median (range))	6 (5–7)	9 (7–10)	< 0.001*^c^
Apgar 5 min < 7 (no. (%))	20 (76.9%)	0	< 0.001*^b^
NICU (no. (%))	26 (100%)	3 (6.8%)	< 0.001*^b^

^*^Significant

^a^Independent *t* test

^b^Chi-square test

^c^Mann–Whitney *U* test

The fetal MPA Doppler measurements in both groups were summarized in Table 3. The mean At/Et ratio and PSV of the fetal pulmonary artery was significantly lower in fetuses that subsequently developed RDS than those without RDS throughout all gestational age period (0.297 ± 0.026 versus 0.352 ± 0.044 and 62.03 ± 4.55 cm s^−1^ versus 66.27 ± 4.43 cm s^−1^, *p* < 0.001) and in the early preterm period (0.280 ± 0.011 versus 0.312 ± 0.028, *p* = 0.002), late preterm period (0.311 ± 0.025 versus 0.362 ± 025, *p* < 0.001), and early term period (0.345 ± 0.021 versus 0.383 ± 0.036, *p* < 0.001). On the other hand, the mean PI, RI of the fetal pulmonary artery were significantly high in fetuses who later developed RDS than in those who did not (2.67 ± 0.188 versus 2.43 ± 0.028, 0.900 ± 0.013 versus 0.882 ± 0.014 respectively, *p* < 0.001).

**Table 3:** Doppler data of fetuses with and without neonatal RDS

	Fetuses with RDS (*n* = 12)	Fetuses without RDS (*n* = 58)	*p* value^a^
All gestational age			
At/Et ratio mean	0.297 ± 0.026	0.352 ± 0.044	< 0.001*
PI mean	2.67 ± 0.188	2.43 ± 0.028	< 0.001*
RI mean	0.900 ± 0.013	0.882 ± 0.014	< 0.001*
PSV mean (cm s^−1^)	62.03 ± 4.55	66.27 ± 4.43	< 0.001*
Early preterm			
At/Et ratio mean	0.280 ± 0.011	0.312 ± 0.028	0.002*
PI mean	2.73 ± 0.19	2.61 ± 0.100	0.036*
RI mean	0.899 ± 0.014	0.883 ± 0.016	0.012*
PSV mean (cm s^−1^)	61.93 ± 4.80	64.64 ± 4.84	0.01*
Late preterm			
At/Et ratio mean	0.311 ± 0.025	0.362 ± 025	< 0.001*
PI mean	2.59 ± 0.13	2.39 ± 0.16	0.006*
RI mean	0.901 ± 0.012	0.883 ± 0.014	0.006*
PSV mean (cm s^−1^)	61.62 ± 4.74	66.17 ± 4.23	0.001*
Early term			
At/Et ratio mean	0.345 ± 0.021	0.383 ± 0.036	< 0.001*
PI mean	2.5 ± 0.14	2.31 ± 0.16	0.158
RI mean	0.903 ± 0.004	0.879 ± 0.122	0.02*
PSV mean (cm s^−1^)	64.5 ± 0.707	68.15 ± 3.78	0.172

^*^Significant

^a^All test was performed by independent *t* test

**Fig. 2:**
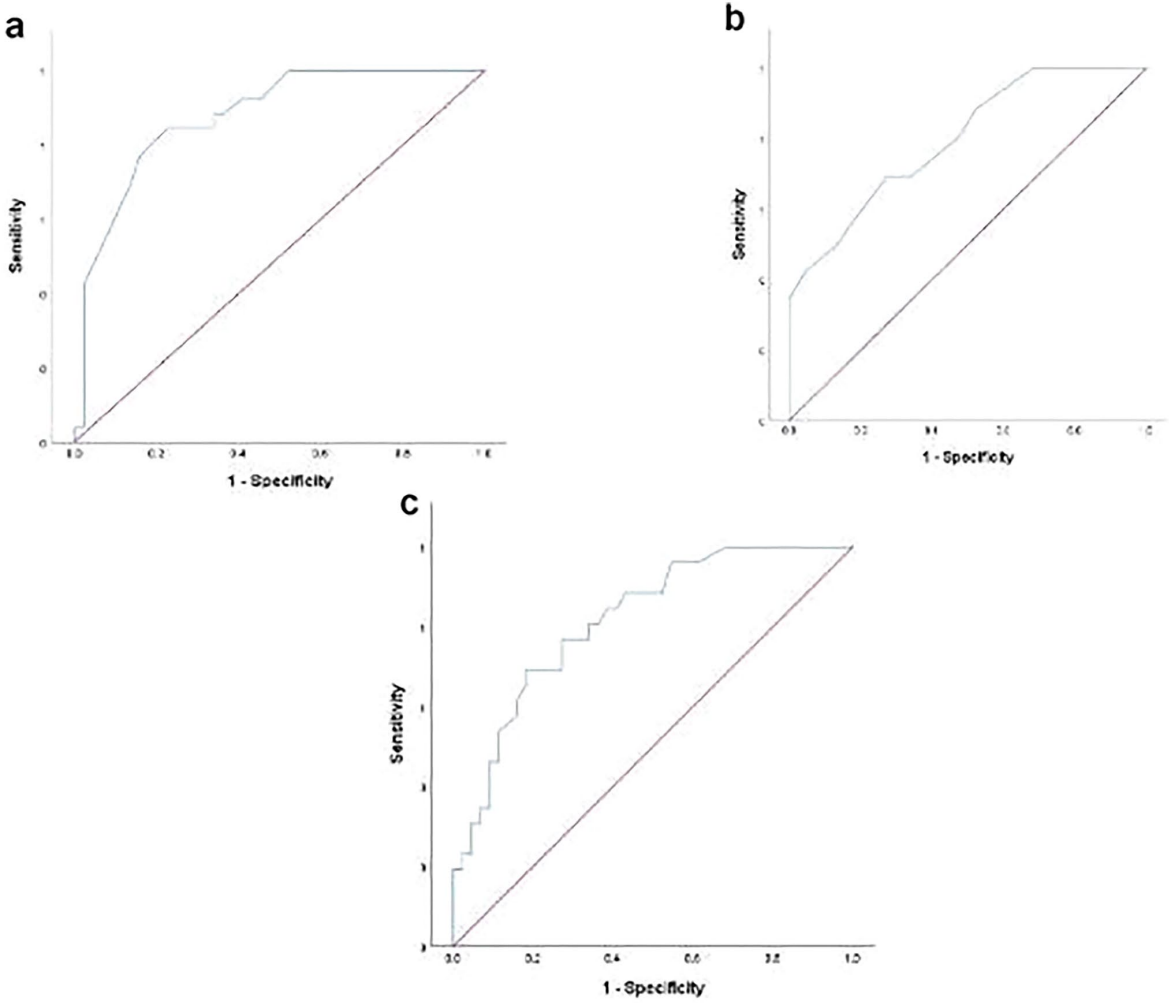
Receiver operating curve (ROC) of the **a** At/Et ratio, **b** PI and **c** RI

The optimal cut off value of At/Et ratio was 0.305 (AUC = 0.878, 95% CI 0.798–0.958, *p* < 0.001) with sensitivity, specificity, PPV and NPV of 76.9, 84.1, 74 and 86%, respectively. on the other hand, PI cutoff value of 2.47 (AUC = 0.797, 95% CI 0.692–0.902, *p* < 0.001) revealed higher sensitivity (80.8%), yet with much lower specificity (52.3%), while the RI revealed cut off value of 0.8962 (AUC = 0.818, 95% CI 0.720–0.916, *p* < 0.001) revealed lower sensitivity (69.2%) and specificity (81.8%) as compared to those of At/Et ratio (Table 4; Fig. 2).

**Table 4:** Cut off value and diagnostic performance of MPA indices

	Cut off	Sensitivity	Specificity	PPV	NPV	AUC	95% CI	*p*
At/Et ratio	0.305	76.9%	84.1%	74%	86%	0.878	0.798–0.958	< 0.001*
RI mean (cm s^−1^)	0.8962	69.2%	81.8%	69.2%	81.8%	0.818	0.720–0.916	< 0.001*
PI mean (cm s^−1^)	2.47	80.8%	52.3%	50%	82.1%	0.797	0.692–0.902	< 0.001*

*Significant

A multivariate logistic regression model reconstructed after adjusting for gestational age revealed that both RI and At/Et ratio significantly predict RDS with odd ratio = 44.07 (*p* < 0.001) and 35.08 (*p* = 0.003) respectively, yet the PI didn’t influence the reconstructed model (Table 5).

**Table 5:** Multinomial logistic regression analysis for RDS prediction of the MPA Doppler indices

	OR	95% CI	*p*
At/Et ratio	35.08	3.30–372.11	0.003*
RI	44.07	4.467–434.853	0.001*
PI	0.451	0.076–2.679	0.381

^*^Significant

## Discussion

Delivery after 38 weeks, markedly decreases the risk of RDS development, as numerable studies have suggested 39 weeks’ gestation as the ideal time for planned caesarian section (CS) [11].

Between 34- and 37 + 6-weeks’ gestation is an area of a possibility of developing RDS, hence an obstetrician should test for fetal lung maturity before attempting to deliver a fetus in this GA range [5].

In our study, fetal MPA Doppler indices for predicting fetal lung maturity and RDS development were examined in preterm and early term fetuses in the third trimester.

Neonatal RDS was diagnosed by neonatologist who was blinded to the fetal MPA Doppler results if at least two out of three from the following criteria were present: (1) clinical signs of respiratory failure including tachypnea, retraction and, or nasal fairing shortly after birth and leading to oxygen requirement for more than one day. (2) Radiographic signs of hyaline membrane disease including: reticulonodular pattern, air bronchogram and ground glass appearance without any other causes of respiratory disease. (3) Clinical response to exogenous pulmonary surfactant.

**Fig. 3:**
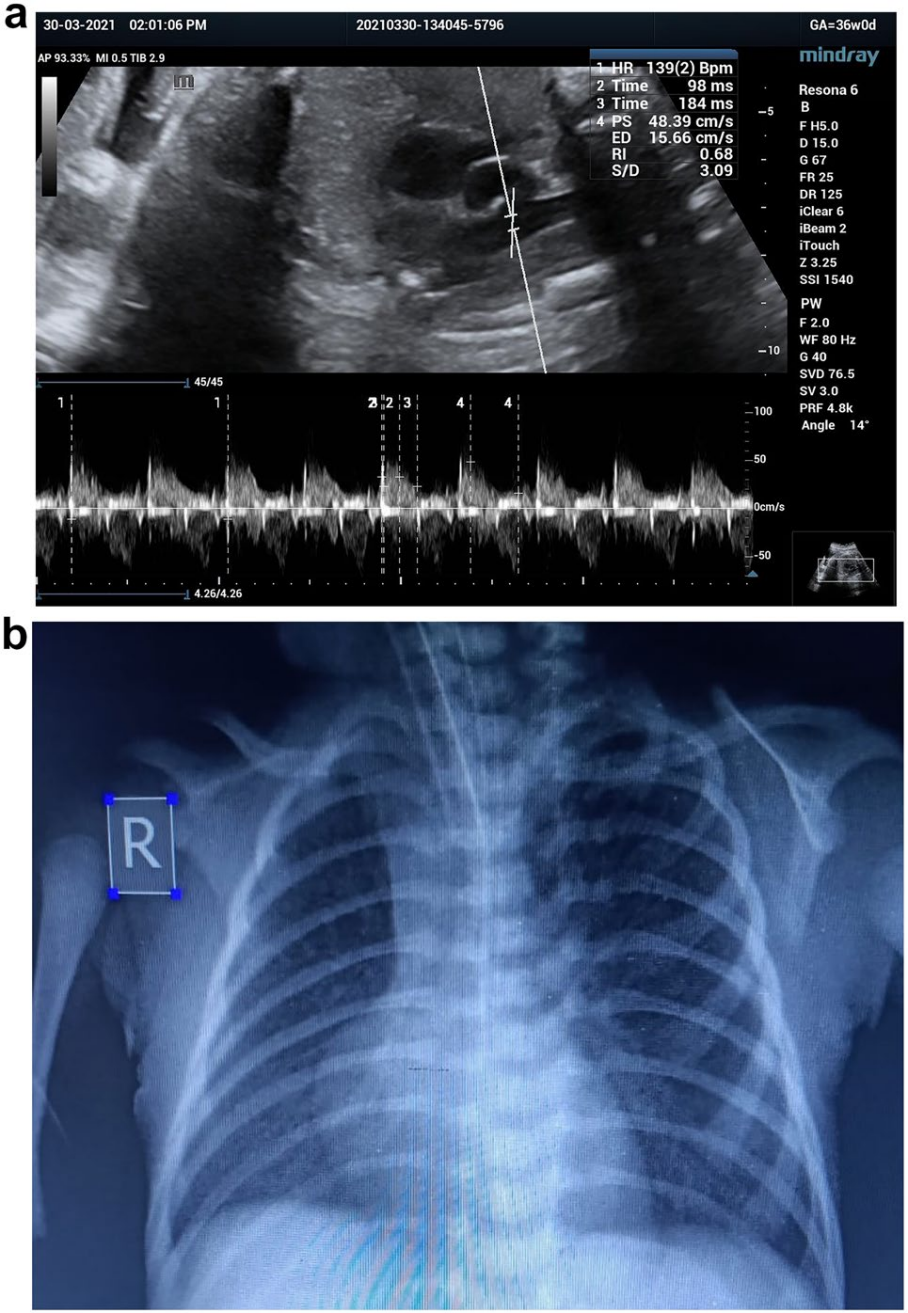
**a** A preterm fetus 34W + 1D with increased all MPA doppler parameter and very low At/Et ratio = 0.16, **b** post-natal chest X-ray shows signs of RDS air bronchograms and ground glass appearance. post-natal 1 min APGAR score was 5 and 5 min was 6, baby was intubated and admitted to NICU

**Fig. 4:**
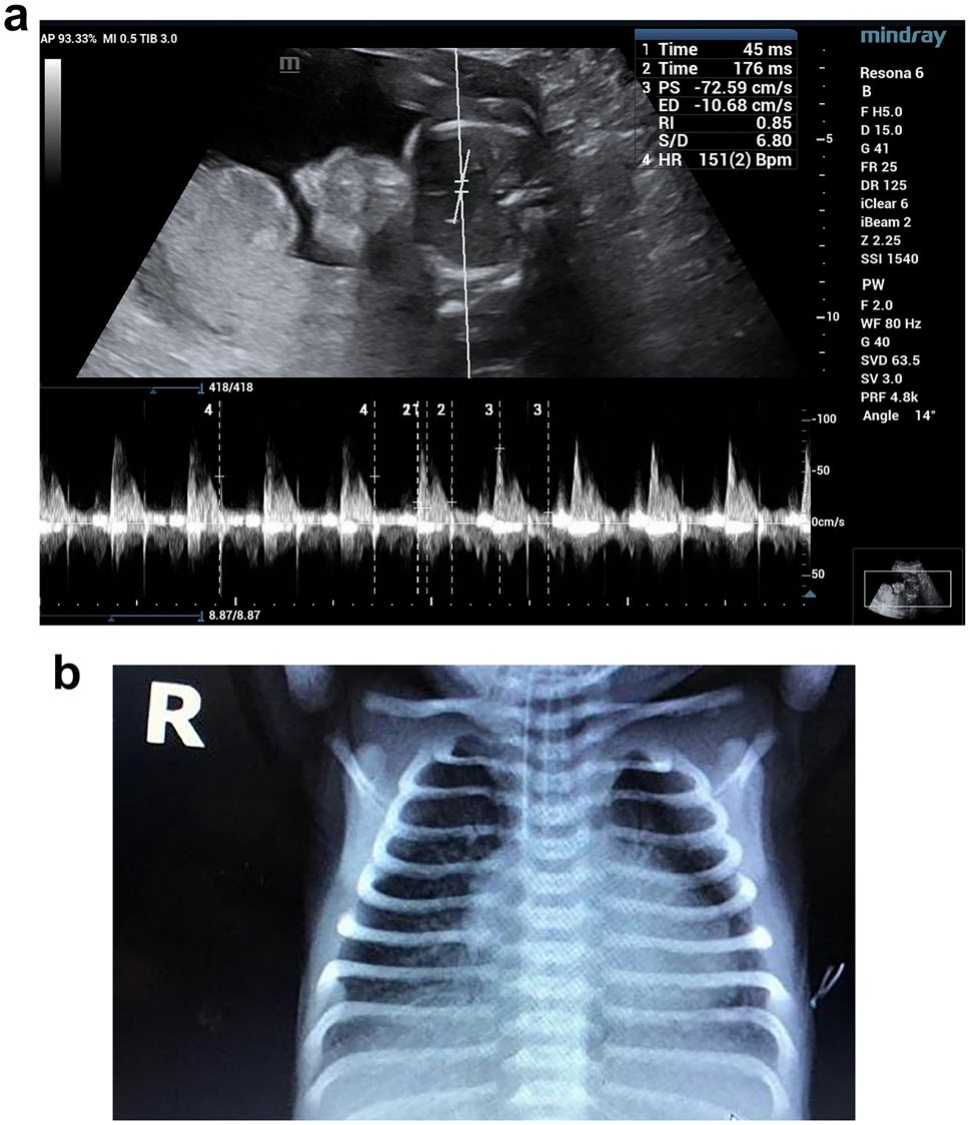
**a** A late preterm fetus 35W + 2D with mild increased all MPA doppler parameter and low At/Et ratio = 0.25, **b** post -natal chest X-ray shows signs of RDS as reticulogranular patterns, air bronchograms. Post-natal 1 min APGAR score was 6 and 5 min was 7, baby was intubated and admitted to NICU

**Fig. 5:**
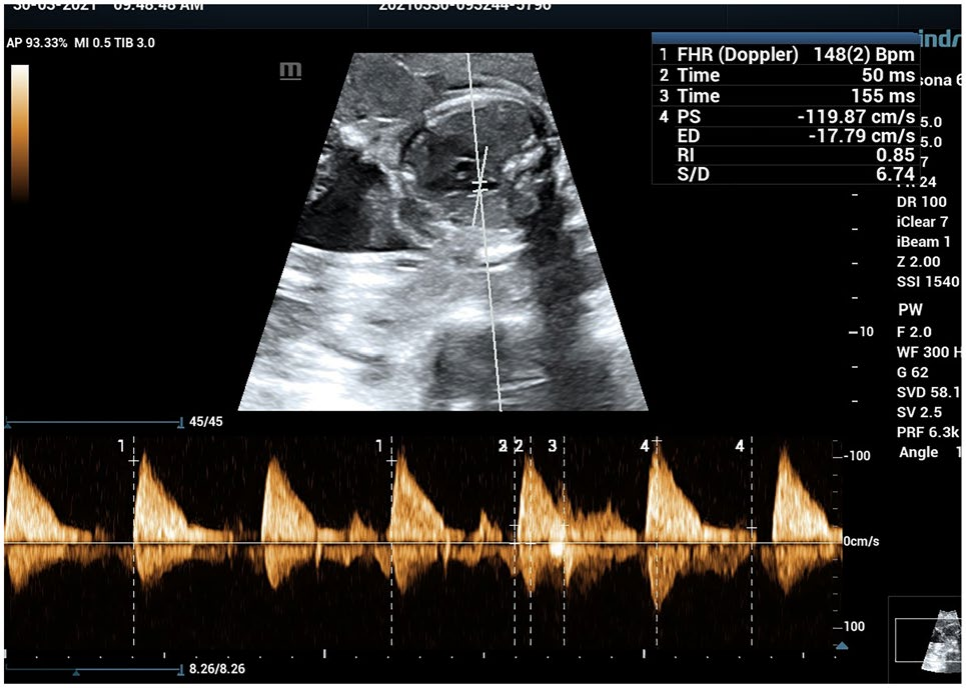
A early term fetus 36W + 5D with average all MPA doppler parameter and high At/Et ratio = 0.38, post-natal 1 min APGAR score was 8 and 5 min was 9

Our study has shown that fetuses that developed RDS had significantly lower At/Et and PSV and higher PI and RI. This may be due to neonates that develop RDS have higher pulmonary vascular resistance and pressure and lower pulmonary blood flow compared with those that do not develop RDS (Figs. 3, 4, 5).

Our study proposes that pulmonary vascular compliance increases and mean pulmonary artery pressure decreases as pregnancy advances, leading to gradual escalation in the pulmonary blood flow. Also [12] have stated that the pulmonary artery impedance decreases as the GA advances.

The inverse relationship between GA and RI is mostly due to the increasing calibre of the pulmonary vessels, increasing vascular elasticity as well as continued pulmonary angiogenesis that eventuate with advancing GA [12].

Similarly [13] reported a significant correlation of gestational age (GA) with At, At/Et, PSV, end-diastolic velocity and mean velocity in 288 healthy fetuses from 22 to 42 weeks’ gestation. MPA At/Et was reported to be have an inverse relationship to the pulmonary artery pressure measured directly at cardiac catheterization. Also, pulmonary artery pressure is well known to be elevated in infants with RDS [12], this makes it possible that MPA At/Et may be useful in prediction of the development of neonatal RDS in preterm births only.

The previously mentioned studies were limited by the relatively small number of patients; also, none had studied the development of RDS in early term fetuses.

In our study, the mean At/Et ratio of the fetal pulmonary artery was remarkably lower in fetuses that subsequently developed RDS than those who didn’t throughout all gestational age period (0.297 ± 0.026 versus 0.352 ± 0.044, *p* < 0.001) and in the early preterm period (0.280 ± 0.011 versus 0.312 ± 0.028, *p* = 0.002), late preterm period (0.311 ± 0.025 versus 0.362 ± 025, *p* < 0.001), and early term period (0.345 ± 0.021 versus 0.383 ± 0.036, *p* < 0.001). This is matching with [7] were the MPA At/Et was significantly lower in fetuses diagnosed with RDS compared with those without being 0.209 ± 0.054 versus 0.332 ± 0.066, *p* < 0.001 respectively. Our results are also in keeping with [15] whose results revealed that At, Et and At/Et ratio are positively correlated with fetal lung maturity, were the mean At/Et ratio was 0.29 ± 0.01 versus 0.34 ± 0.03 (*p* value < 0.001) in RDS (+) and RDS (−) foetuses respectively (14). Unlike the study done by [5] the mean AT/ET ratio value was higher in neonates who developed RDS (0.24 ± 0.07) as compared to those that did not (0.18 ± 0.04).

The optimal cut off value of At/Et ratio in our study was 0.305 (AUC = 0.878, 95% CI 0.798–0.958, *p* < 0.001) with sensitivity, specificity, PPV and NPV of 76.9, 84.1, 74 and 86% respectively. This is in keeping with [7] were the cut off value of the At/Et ratio is 0.305 with sensitivity 76.4% and specificity 91.6% and AUC 0.899.

This is also matching with [15] in which ratio cut point was 0.304 by which RDS (–) can be detected with high sensitivity, specificity, positive predictive value and negative predictive value 94.3, 95.7, 99.1and 75.9% respectively. The cut off value according to [5] was 0.22 providing a sensitivity of 61.5%, specificity of 88%, NPV of 95.9% and PPV of 33.3% (*p* = 0.0023).

Kim et al. [9] compared the fetal MPA Doppler measurements with post-natal clinical data as regards the development of fetal RDS with cut point of At/Et ratio 0.326 for predicting RDS, and claimed that ratio more than 0.326, means subsequent development of RDS. Their results are of opposite to the results of [8] and [13] and ours.

In a study done by [8] MPA At/Et and the TDx -FLM-II (measured in the amniotic fluid) were positively correlated, which means that an increased At/Et is associated with a more mature lung and a lower risk of RDS development, which supports our findings. However they didn’t correlate the results with the post-natal clinical data for RDS.

On the other hand, Azpurua et al. [14] have concluded that At/Et has an inverse relationship with lecithin/sphingomyelin ratio obtained by amniocentesis. However, their study could not comment on the association of At/Et with the clinical development of RDS due to their relative small study sample size (29 fetuses) including only a single case diagnosed with RDS.

Alternately, the mean MPA PI and RI of the fetal pulmonary artery were remarkably high in fetuses who later developed RDS than in those who did not (2.67 ± 0.188 versus 2.43 ± 0.028, 0.900 ± 0.013 versus 0.882 ± 0.014 respectively, p < 0.001), while PSV was remarkably lower in fetuses suffering from RDS (62.03 ± 4.55 cm s^−1^ versus 66.27 ± 4.43 cm s^−1^, *p* < 0.001). This is in keeping with [7] were the MPA PI and RI were remarkably higher (2.27 ± 0.23 and 0.8 ± 0.11 cm s^−1^ versus 2.18 ± 0.23 and 0.76 ± 0.09 cm s^−1^; *p*: 0.003 and 0.002, respectively), whereas PSV was significantly lower in fetuses with RDS (65.05 ± 5.33 cm s^−1^ versus 67.21 ± 4.8 cm s^−1^; *p*: 0.002). Our results are also matching with [6] were the MPA PI and RI were remarkably higher in fetuses who developed RDS as compared to those with who didn’t (2.51 ± 0.33 and 0.90 ± 0.03 cm s^−1^ versus 1.96 ± 0.20 and 0.84 ± 0.01 cm s^−1^; *p* value < 0.001 and < 0.001 respectively). However, according to [15] other Doppler indices including RI, PI, PSV and S/D were not different between RDS (+) and RDS (−) fetuses.

In our study the PI cut off value of 2.47 (AUC = 0.797, 95% CI; 0.692–0.902, *p* < 0.001) revealed higher sensitivity (80.8%), yet with much lower specificity (52.3%), while the RI revealed cut off value of 0.8962 (AUC = 0.818, 95% CI; 0.720–0.916, *p* < 0.001) revealed lower sensitivity (69.2%) and specificity (81.8%) as compared to those of At/Et ratio. This is in keeping with [6] study were the optimum cut-off was 2.33 for MPA PI (AUC = 0.96; *p* < 0.001) with accuracy 94.4% while the MPA RI was 0.89 for (AUC = 0.94; *p* < 0.001with accuracy 88.1%.

The limitation of our study is the relatively small sample size. A larger number of patients will allow better assessment of its accuracy in predicting neonatal outcomes.

In conclusion, fetal MPA Doppler measurements especially MPA RI and At/Et ratio significantly predict RDS after adjusting for gestational age and provide a non-invasive means of detecting fetal lung maturity with high sensitivity, specificity, and predictive values. This modality can be applied clinically and thus decreasing the need for amniocenteses in the future.

## Data Availability

Available on request with the corresponding author. The authors declare that they had full access to all data in this study and the authors take complete responsibility for the integrity of the data and the accuracy of the data analysis.

## Abbreviations

PI: Pulsatility index
RI: Resistance index
PSV: Peak systolic velocity
RDS: Respiratory distress syndrome
GA: Gestational age
At/Et ratio: Acceleration time/ejection time
MPA: Main pulmonary artery
AUC: Area under curve
DM: Diabetes mellitus
HTN: Hypertension
PROM: Premature rupture of membrane
CS: Caesarian section
TDx-FLM-II: TDx fetal lung maturity

## Declarations

### Permission to reproduce material from other sources

No material used from other sources.
